# Three years follow-up of Venetoclax in advanced-stage, relapsed or refractory AL amyloidosis with cardiac involvement and t(11;14) with BCL2 expression

**DOI:** 10.1007/s00277-024-05901-x

**Published:** 2024-07-17

**Authors:** Max J. Rieger, Thomas Pabst, Barbara Jeker, Pamella Paul, Fabio Bergamini, Marco M. Bühler, Adalgisa Condoluci, Andreas J. Flammer, Davide Rossi, Georg Stussi, Bernhard Gerber, Rahel Schwotzer

**Affiliations:** 1 Department of Medical Oncology and Hematology, University and University Hospital of Zurich, Rämistrasse 100, 8091 Zurich, Switzerland; 2 Department of Medical Oncology, Inselspital, Bern University Hospital, Bern, Switzerland; 3 Clinic of Hematology, Oncology Institute of Southern Switzerland, Ente Ospedaliero Cantonale, Bellinzona, Switzerland; 4 Department of Pathology and Molecular Pathology, University Hospital and University of Zurich, Zurich, Switzerland; 5 Laboratory of Experimental Hematology, Institute of Oncology Research, Bellinzona, Switzerland; 6 University Heart Center, University Hospital Zurich, Zurich, Switzerland; 7 Faculty of Biomedical Sciences, Università Della Svizzera Italiana, Lugano, Switzerland; 8 University of Zurich, Zurich, Switzerland

**Keywords:** Venetoclax, AL Amyloidosis, Revised Mayo Stage III/IV, t(11;14), BCL-2

## Abstract

Further line treatment of patients with advanced stage AL amyloidosis with cardiac involvement is challenging. Venetoclax is a promising option, especially in t(11;14) and BCL2 expression.

In our multicentre observational study, we report the 3-year follow-up of Venetoclax treatment in 9 patients with advanced, relapsed or refractory AL amyloidosis with t(11;14) and BCL-2 expression in > 50% of plasma cells. At baseline, all patients had been previously treated with daratumumab, all had cardiac involvement with revised Mayo stage III or IV/ European modification of Mayo 2004 IIIA or IIIB (1/9 unclassified due to missing troponin T), 5/9 patients had renal involvement.

After a median of 35 months (range 25–49) since the start of Venetoclax, 8/9 patients were still alive (OS 89%). First and best hematological responses were observed after a median of 26 days (11–125) and 106 days (35–659), overall response rate was 100% (7/9 CR, 2/9 VGPR). Where observed, organ response was documented within the first 6 months of therapy, including cardiac (6/9) and renal (3/5) improvements. Venetoclax was discontinued in 6/9 patients after a median of 15 months (11–48) due to toxicity (2/9), disease progression (2/9), fixed treatment duration (1/9), or safety concerns (1/9).

In conclusion, Venetoclax induces a rapid and deep hematologic response with consistent improvement in organ function with an acceptable safety profile in patients with pretreated, advanced stage AL amyloidosis with cardiac involvement and BCL2 expression with and potentially without detected t(11:14), which warrants further investigation.

**Supplementary Information:**

The online version contains supplementary material available at 10.1007/s00277-024-05901-x.

## Introduction

Immunoglobulin light chain amyloidosis (AL amyloidosis) is the most common form of amyloidosis alongside wild-type transthyretin amyloidosis [1, 2]. The course of the disease largely depends on the severity of organ involvement [3]. Patients with heart involvement and high light chain burden have demonstrated inferior outcome [4]. The phase 3 ANDROMEDA trial established a new standard-of-care with the combination of daratumumab and VCD (bortezomib, cyclophosphamide and dexamethasone) followed by an 18 months daratumumab maintenance in newly diagnosed patients with European modified Mayo 2005 stage I—IIIA AL amyloidosis. The trial has redefined cornerstones of first-line therapy in a non-transplant setting for such patients [5], as the results suggested an impressive overall haematological response rate of 92% and high organ response rates at 6 and 12 months (cardiac response rates of 41.5% and 57%, renal response of 53% and 57%) leading to the approval of this combination [6].

However, treatment of patients with relapsing or refractory AL amyloidosis, especially in advanced stages (≥ IIIA), remains challenging. Relevant impaired organ function often limits common treatment options, such as immunomodulatory therapy with IMiDs, which are often associated with further deterioration of renal function and an increase in NT-proBNP, a surrogate marker of cardiac function [7–10].

Interestingly, plasma cells in patients with AL amyloidosis differ from plasma cells in patients with plasma cell myeloma (PCM) [11]. In AL amyloidosis, plasma cells are more susceptible to apoptosis and appear to be particularly dependent on the anti-apoptotic proteins MCL-1 and BCL2 [12]. Associated with an BCL2 expression, the most frequently observed recurrent cytogenetic abnormality by interphase fluorescence in situ hybridization (iFISH) in patients with AL amyloidosis (40–50%) is t(11;14), more frequently than in patients with PCM (15–20%) [13]. The BCL2 inhibitor Venetoclax has shown promising results in early reports of patients with AL amyloidosis with t(11;14) [14, 15]. In retrospective studies, the overall hematological response rate according to the ISA amyloidosis response criteria was as high as 88% (66.6—88%) [16–19]. In a retrospective study by Lebel et al. including 26 patients followed for a median of 33 months, overall survival was 77%. However, detailed information on potential predictive biomarkers of Venetoclax treatment response, such as BCL2 expression, as well as details on organ response are lacking in all published studies reporting on Venetoclax, likely due to the mostly short follow-up period and the retrospective nature of the studies [20].

We previously reported the haematological response to Venetoclax in nine patients with advanced stage amyloidosis from the Swiss Amyloidosis Registry (SAR) with t(11;14) in iFISH and BCL2 expression. Here, we report on a three-year follow-up including the treatment schedule, the time to and the depth of hematologic and organ responses, as well as adverse events and reasons for treatment discontinuation.

## Material and Methods

In this retrospective observational study involving three centers of the Swiss Amyloidosis Network, patients > 18 years of age with relapsed or refractory AL amyloidosis and t(11;14) and BCL2 expression in over 50% of plasma cells were enrolled between June 2018 and December 2021. Expression of BCL2 in bone marrow plasma cells was evaluated using immunohistochemical (IHC) staining (BCL2), with high expression defined as > 50% of plasma cells with moderate or high intensity, in analogy to the Bellini trial [21](Supplemental Fig. 1). The t(11;14) signature was detected on iFISH studies of the bone marrow plasma cells.

**Fig. 1:**
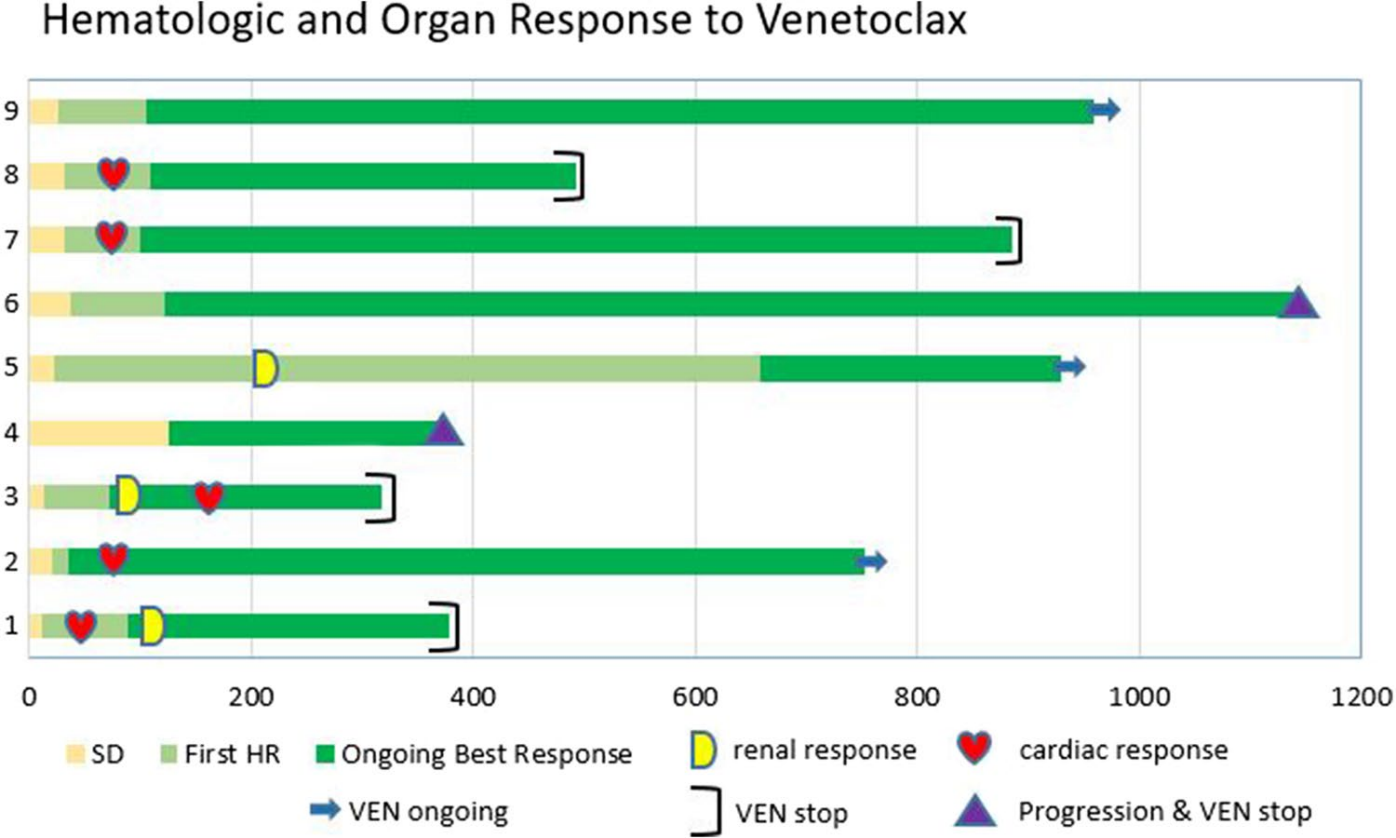
Hematologic and organ response over time in days from start of Venetoclax (VEN) Legend; SD: Stable Disease, HR; Hematologic Response, VEN: Venetoclax, Hem: Hematologic

Follow-up data of the patients were collected as part of the routine patient care within the Swiss Amyloidosis Registry (SAR), a prospective longitudinal data registry involving an increasing number of sites in Switzerland [2]. All patients gave written informed consent and the SAR was approved by a local ethics committee (PB_2016_01744). The competent local ethics committee waived the need for formal review board approval of the present substudy (Req-2022–02088). End of follow-up of this project was December 31st 2023.

Diagnosis of systemic AL amyloidosis required biopsy proven light-chain amyloid deposits with typical green birefringence under cross-polarized light following Congo red staining and positive immunohistochemistry [22]. Baseline data were captured at the time of Venetoclax treatment initiation. Organ involvement was defined as biopsy proven amyloid deposit of the concerning organ (or tissue) and/or typical organ alterations as defined for AL amyloidosis [22]. Hematological response and organ response were recorded according to the currently accepted response criteria [23, 24]. Organ function and hematological parameters at diagnosis served as the reference values. Primary outcome was overall survival, secondary endpoints were hematological response, organ response and treatment-related adverse events. Statistical analysis and graphical figures were perfomed using *R Studio software* (version 4.3.2.) and Graphpad Prism (version 10.0.0).

## Results

### Patient characteristics and diagnosis

Table 1 shows baseline characteristics of patients including previous treatment before Venetoclax. At treatment initiation, patients had a median age of 67 years (range 61 – 89 years) and a median number of 3 previous lines of therapy (range 1–4), all including daratumumab. Other therapies included high-dose melphalan (200 mg/m2) with autologous stem cell transplantation (aSCT, 2/9 patients), bortezomib (6/9), and lenalidomide (5/9). Median time from diagnosis to Venetoclax initiation was 40 months (range; 3 to 105 months).

**Table 1:** Baseline Characteristics at Diagnosis / Start of VEN

Pat	Age	Sex	BM PC % (clonality)	iFISH	Organ involvement	dFLC	Risk Stage^a^	BCL-2 expr (%)	eGFR (CKD-EPI)	Uprot/crea (mg/mmol)	(N.o.) Prior Tx (best HR)	Time to VEN (d)
1	63	F	15 (λ)	t(11;14), + 11q, -14q	Heart, Kidney (glom), PNS, GI	223.3	IV/ IIIa	> 50	95	299	(1) Dara-VD (PR)	87
2	67	M	10 (λ)	t(11;14)	Heart, Kidney (vasc), PNS	678	IV/ IIIa	> 50	65	17	(1) Dara-VD (PR)	105
3	89	F	10 (κ)	t(11;14),—3’IGH	Heart, Kidney (glom), PNS, GI	121.8	*NA*	> 90	63	134	(4) VCD, MelD, RD, Dara (VGPR)	477
4	80	F	10 (λ)	t(11;14), -13q14, + 1q	Heart	338	IV/ IIIa	> 50	41	26	(1) Dara-VMP (VGPR)	487
5	82	M	20 (λ)	t(11;14)	Heart, PNS, ANS, Kidney (vasc)	236.4	IV/ IIIa	> 90	37	14	(2) MelD, DaraD (PR)	1186
6	61	M	10 (λ)	t(11;14), -13	Heart, Kidney, Skin	882	III/ IIIa	> 50	38	38	(4) VCD, aSCT, RD, Dara (CR)	3145
7	66	F	20 (λ)	t(11;14)	Heart	115	III/ IIIb	> 90	68	*NA*	(3) VCD, Rd, Dara (VGPR)	2330
8	72	F	15 (λ)	t(11;14); + 3; + 5; + 7; + 13; + 15; + 19	Heart	88	III/ IIIb	> 50	70	*NA*	(3) VCD, Rd, Dara (VGPR)	1846
9	61	M	10 (λ)	t(11;14)	Heart	122	III/ IIIa	> 90	67	*NA*	(3) VCD, aSCT, Dara-Rd (CR)	2458

Legend: *aSCT* Autologous stem cell transplantation, *BM* Bone marrow, *CR* Complete response, *dFLC* Difference in free light chain ratio, *Pat* Patient, *eGFR* Estimated glomerular filtration ratio, *expr* expression, *glom* Glomerular type, *GI* Gastrointestinal; *HR* Hematologic response, *iFISH* Interphase fluorescence-in-situ-hybridisation, *N.o.* Numbers of, *PC* Plasma cells, *PR* Partial remission, *PNS* Peripheral nervous system, *Tx* Therapies, *vasc* Vascular type, *VEN* Venetoclax, *VGPR* Very good partial remission

^a^risk stage according to the revised Mayo risk model/ Europ modification of Mayo 2004

All patients had advanced cardiac involvement according to the revised Mayo risk model, 4/9 patients had stage IV disease and 4/9 patients had stage III disease; according to the European modification of the 2004 Mayo model, 6/9 had stage IIIA disease and 2/9 had stage IIIB disease [4, 25]. 1/9 patient was unclassifiable due to a missing troponin T measurement at baseline. Renal involvement was present in 5/9 patients; potential renal involvement was not detected or not clinically relevant in 4/9 patients. At baseline, all patients had estimated glomerular filtration rate (eGFR) of > 30 ml/min/173m^2^ (range 37 – 95).

The median bone marrow (BM) plasma cell infiltration was 10% (range 10–20%), no patients had concurrent symptomatic myeloma diagnosis. All histological samples showed medium to strong intensity BCL2 expression in > 50% of plasma cells, and had t(11;14) demonstrated by iFISH. The median difference between involved and non-involved free light-chains (dFLC) at baseline was 223 mg/L (range 88–678 mg/L), and median NT-proBNP level was 3625 pg/ml (range 652–10′352 pg/ml).

### Venetoclax treatment

Oral Venetoclax was given in combination with bortezomib and dexamethasone (4/9), as monotherapy (3/9), with dexamethasone (1/9), or combined with ongoing daratumumab (1/9). The final dose of Venetoclax was 400 mg (7/9), 600 mg (1/9) and 800 mg (1/9), according to institutional policy. All patients had an initial ramp-up phase (7/9 over three days, 2/9 over three weeks) and received anti-infective prophylaxis with valacyclovir and trimethoprim/sulfomethoxazole. Intravenous immunoglobulins were regularly replaced in 5/9 patients. Adherence to therapy was incomplete in two patients (one VGPR, and one PR).

Tumour lysis prophylaxis was initiated with allopurinol in 8/9 patients and with rasburicase in 4/9 patients before the start of therapy.

### Response, outcome and end of treatment

Median follow-up from Venetoclax start was 35 months (range 25–49). At last follow-up, 8/9 patients were still alive (89%). 6/9 patients discontinued Venetoclax after a median duration of therapy of 15 months (range 11–48).

**Fig. 2:**
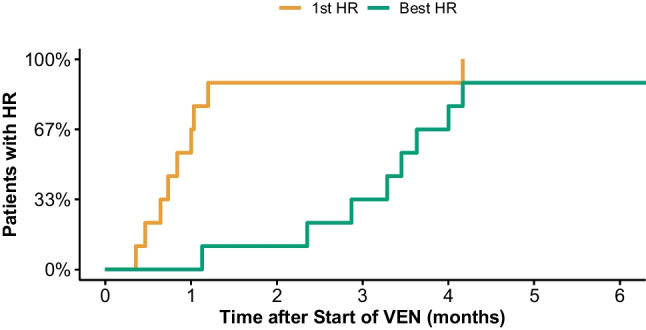
Time to First Hematologic Response (1st HR) and Best Response (Best HR) after Venetoclax (VEN) Initiation

The evolution of hematologic and organ response over time is shown in Fig. 1. After treatment initiation, first and best hematological responses were observed after a median of 26 days (range 11–125 days), and 106 days (range 35–659 days), respectively (Fig. 2). The hematological overall response rate was 100% with 7/9 achieving CR and 2/9 achieving VGPR as their best remission status (Table 2).

**Table 2:** Characteristics of Response to Venetoclax

Pat	HR pre-Ven	VEN combo	VEN days	Target mg/d (ramp up duration)	AE Cytopenia (CTCAE)	Other AE (CTCAE)	Days to First HR (Type)	Days to Best HR (Type)	dFLC red. (%)	Organ resp. (best)	NTpBNP red. (%)	Reason for EoT
1	PR	VEN	378	800 (3d)	-	Infection (G2)	11 (VGPR)	88 (CR)	-96	Heart (VGPR), Kidney (VGPR)	- 80	Fixed duration
2	PR	VEN + D	753	600 (3d)	-	Tremor (G2), Infection (G2), heart failure (G2)	20 (VGPR)	35 (CR)	-97	Heart (PR)	-66	*NA*
3	PR	VEN + Dara	317	400 (4w)	Hb (G3)	GI (G1)	14 (VGPR)	72 (CR)	-97	Heart (VGPR), Kidney (CR)	-78	Safety concern
4	PR	VEN	372	400 (3d)	ANC (G2)	GI (G1)	125 (VGPR)	125 (VGPR)	-77	-	-12	Hem Progress
5	SD	VEN	930	400 (4w)	-	Infection (G2)	22 (PR)	659 (CR)	-99	Kidney (PR)	none	*NA*
6	PD	VEN + VD	1148	400 (3d)	ANC (G2), Hb (G2), Tc (G2)	PNP (G2); Diabetes (G1), GI (G2)	37 (PR)	122 (VGPR)	-90	-	none	Hem Progress
7	PD	VEN + VD	885	400 (3d)	ANC (G3), Hb (G3), Tc (G3)	Infection (G2)	31 (PR)	100 (CR)	-99	Heart (PR)	-65	AE, Adheren
8	PD	VEN + VD	492	400 (3d)	ANC (G2), Hb (G2), Tc (G2)	PNP (G2), GI (G2), Infection (G2)	31 (VGPR)	109 (CR)	-97	Heart (PR)	-71	AEs (Infection)
9	PD	VEN + VD	960	400 (3d)	ANC (G4); Hb (G3), Tc (G4)	PNP (G2), Diarrhea (G1)	26 (PR)	106 (CR)	-99	Heart (PR)	-52	*NA*

Legend: *Pat* Patient, *AE* Adverse events, *ANC* Absolute neutrophil count, *CTCAE* Common Terminology Criteria for Adverse Events, *CR* Complete remission, *dFLC* Difference in free light chain ratio, *EoT* End of Treatment, *GI* Gastrointestinal, *G* Grade, *Hb* Hemoglobin, *HR* Hematologic remission, *NTpBNP* N-Terminal pro-Brain-Natriuretic Peptide, *PNP* Polyneuropathy, *PR* Partial remission, *red.* Reduction, *resp.* Response, *Tc* Thrombocyte count, *VEN* Venetoclax, *VGPR* Very good partial remission

**Fig. 3:**
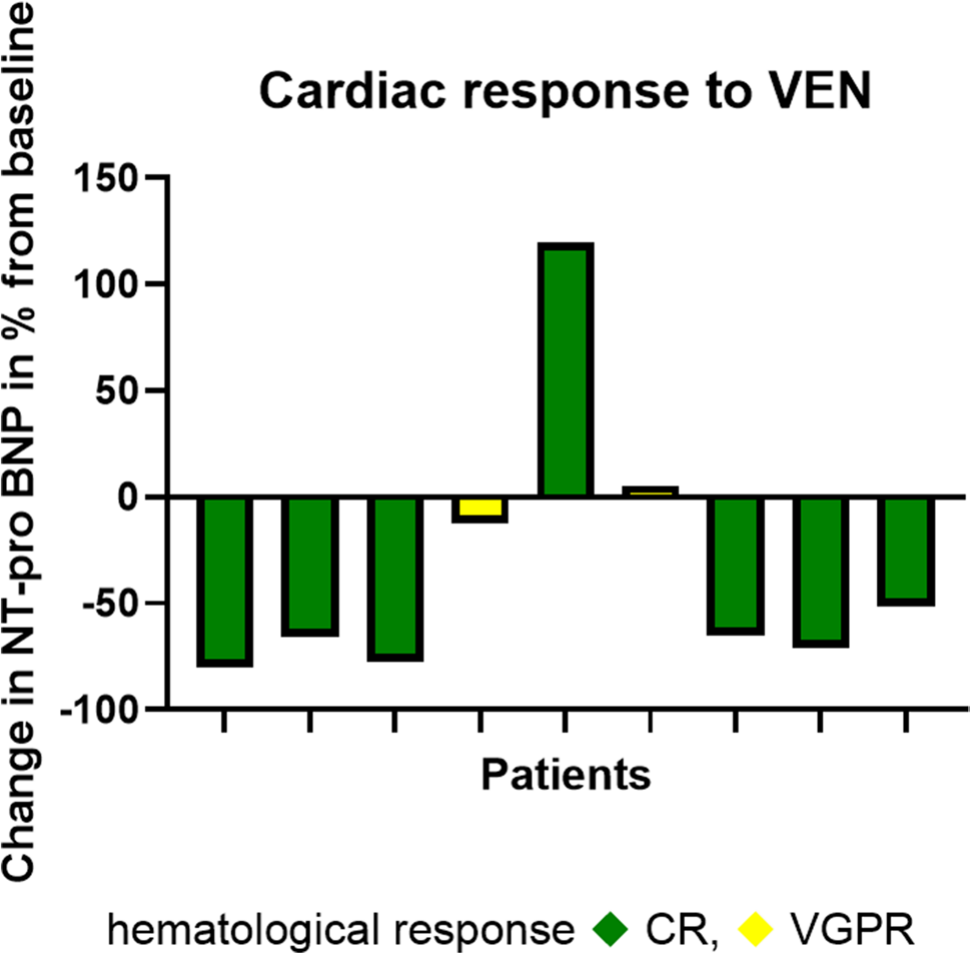
Change of NT-pro BNP from Baseline after Venetoclax initiation in patient 1 to 9 (from left to right)

Cardiac response was observed in 6/9 patients and renal response was observed in 3/5 affected patients. The median best NT-proBNP reduction from baseline was—65% (range -80% to + 119%) as shown in Fig. 3.

Of all patients, 2 showed hematologic progression during Venetoclax treatment (after 372 and 1148 days) while 4/6 patients discontinued Venetoclax for other reasons. Other reasons for discontinuation were toxicity/lack of adherence (2/6 patients) and fixed treatment duration treatment (1/9). 1/9 was stopped in 2019 for safety concern due to the interim results of the BELLINI trial [21] (Table 2).

### Adverse events

Overall, the initial tolerability of Venetoclax was relatively good in all patients. The most common adverse events accumulating over time were primarily hematologic toxicity (6/9 patients with anaemia CTCAE grade 2–3, neutropenia grade 2–4, and/or thrombocytopenia up to grade 2). Gastrointestinal toxicities were observed in 5/9 patients and were mostly mild (grade 1). In 4/9 patients, recurrent infections (min. grade 2) complicated Venetoclax treatment and eventually lead to treatment discontinuation in 2 patients. 4/9 patients (all on Dara-VD) experienced peripheral nervous system toxicity, concomitant Bortezomib was therefore omitted in 2/9 patients after two cycles. None of the patients experienced relevant laboratory or clinical tumour lysis. Dose reduction of Venetoclax was performed in 1/9 patients due to recurrent infections without significant neutropenia (800 to 600 mg/d).

## Discussion

The results of our multicenter observational study demonstrate that Venetoclax can induce rapid, deep and durable hematologic responses, resulting in a clinically meaningful, consistent improvement in organ function, in patients with relapsed/refractory AL amyloidosis with t(11;14) and BCL2 expression.

Despite the small number in our cohort and the retrospective nature, this study provides new insights into this highly vulnerable patient population with advanced age (median 67 years) and advanced disease stages with cardiac involvement with Revised Mayo/Modified European Mayo 2004 stage IIIA and IIIB/ III and IV disease, where treatment options are generally limited.

The remarkable overall response rate, rapidity and quality of response observed in this cohort could potentially be explained by the ubiquitous presence of t(11;14) and the associated BCL2 expression. As previously shown, the presence of t(11;14) is present in up to 40–60% of AL amyloidosis patients and is generally associated with a favourable response to Venetoclax in both patients with AL amyloidosis and plasma cell myeloma [21, 26]. The associated BCL2 expression demonstrated here may serve as an alternative surrogate for potential treatment efficacy, as suggested previousely [21, 27]. This would be of particular importance inpatients with low plasma cell infiltration where iFISH is unavailable. However, this theory requires further investigation as we did not includ patients without documented t(11:14).

Our results are particularly valuable as they include patients with a relatively high disease burden with a baseline bone marrow plasma cell infiltration rate of ≥ 10% and a median dFLC of 223.3 mg/l (range 88–882 mg/l), all pretreated with standard-of-care daratumumab.

Even in this advanced and highly vulnerable population, Venetoclax demonstrated an immediate benefit with relatively good tolerability. The overall haematological response of 100% (VGPR/CR) and the median time to first response of 26 days is similar to that reported by others [16, 18, 20]. Unlike others, we were nonetheless able to demonstrate a subsequent cardiac improvement with an impressive reduction in NT-proBNP in most patients. Presumably, this is also reflected in the high overall survival rate of 89% after 3 years.

To further improve the treatment of AL amyloidosis, Venetoclax needs to be carefully evaluated in prospective trials to optimize tolerability, dosing, treatment duration and combination therapy. Fixed duration schedules (e.g. 6–12 months), daily Venetoclax doses ≤ 400 mg, steroid sparing regimens as well as combination therapies with newer drugs could enhance the safety profile of Venetoclax in AL amyloidosis and mitigate some of the side effects observed in our series, such as cytopenia, infectious diseases, and peripheral neuropathy.

In conclusion, Venetoclax demonstrates an acceptable safety profile and induces a rapid and deep hematologic response with improved organ function in patients with pretreated, advanced-stage AL amyloidosis with cardiac involvement and t(11;14). However, prospective trials are warranted to draw definitive conclusions.

## Supplementary Information

Supplementary Material 1: BCL2 expression in bone marrow plasma cells (red arrows) by immunohistochemistry.

## Data Availability

All data can be made available upon reasonable request to the corresponding author.

## Declarations

### Permission to reproduce material from other sources

N/a.

### Clinical trial registration

N/a.
