# Supplementary figures and images for: Three years follow-up of Venetoclax in advanced-stage, relapsed or refractory AL amyloidosis with cardiac involvement and t(11;14) with BCL2 expression
Source: Ann Hematol. 2024 Jul 17;103(10):4163–70. doi: 10.1007/s00277-024-05901-x (PMC11512835; doi:10.1007/s00277-024-05901-x)

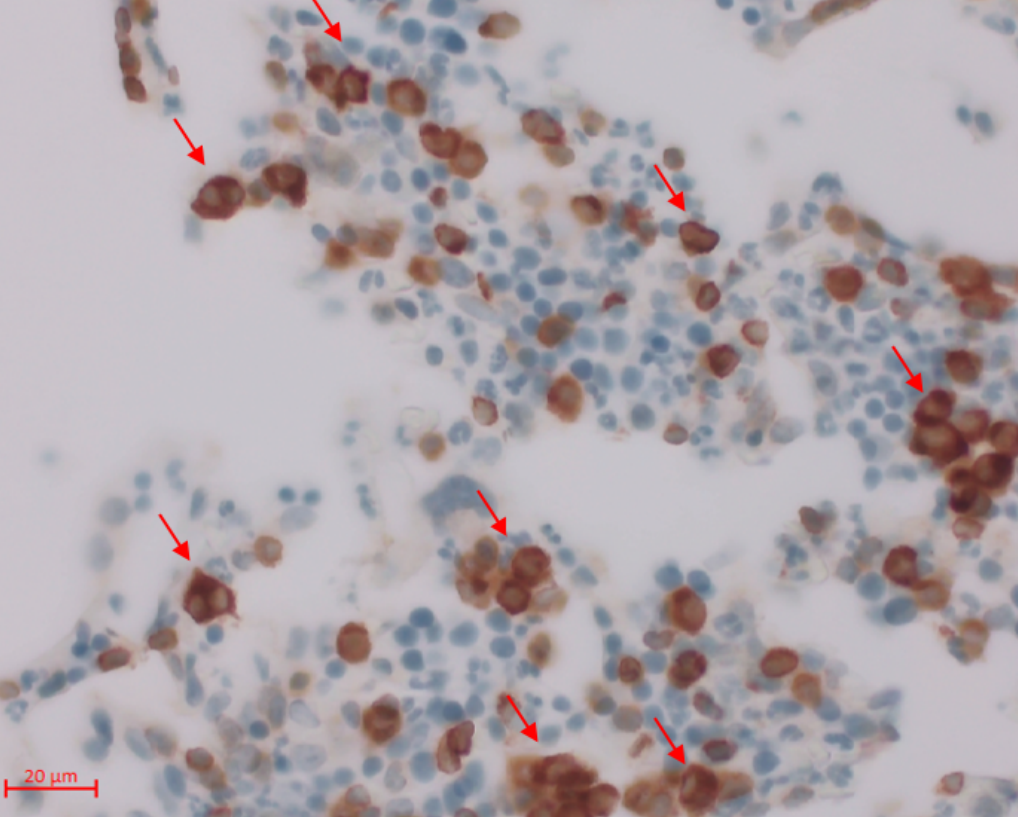

Supplement: Supplementary file 1 — Supplementary Material 1: BCL2 expression in bone marrow plasma cells (red arrows) by immunohistochemistry. [file 277_2024_5901_MOESM1_ESM.tif]
